# The gut microbiota predicts and time-restricted feeding delays experimental colitis

**DOI:** 10.1080/19490976.2025.2453019

**Published:** 2025-01-22

**Authors:** Hannah K. Ruple, Eva Haasis, Anna Bettenburg, Carina Maier, Carolin Fritz, Laura Schüle, Sarah Löcker, Yvonne Soltow, Lynn Schintgen, Nina S. Schmidt, Celine Schneider, Axel Lorentz, W. Florian Fricke

**Affiliations:** a Department of Microbiome Research and Applied Bioinformatics, Institute for Nutritional Sciences, University of Hohenheim, Stuttgart, Germany; b Institute of Nutritional Medicine, University of Hohenheim, Stuttgart, Germany; c Institute for Genome Sciences, University of Maryland School of Medicine, Baltimore, MD, USA

**Keywords:** Biological clock, gut microbiota, circadian rhythm, time-restricted feeding, experimental colitis, IL-10, inflammatory bowel disease, IBD, colitis prediction

## Abstract

The etiology of inflammatory bowel disease (IBD) remains unclear, treatment options unsatisfactory and disease development difficult to predict for individual patients. Dysbiosis of the gastrointestinal microbiota and disruption of the biological clock have been implicated and studied as diagnostic and therapeutic targets. Here, we examine the relationship of IBD to biological clock and gut microbiota by using the IL-10 deficient (*IL-10*^*-/-*^) mouse model for microbiota-dependent spontaneous colitis in combination with altered (4 h/4 h) light/dark cycles to disrupt and time-restricted feeding (TRF) to restore circadian rhythmicity. We show that while altered light/dark cycles disrupted the intestinal clock in wild type (WT) mice, *IL-10*^*-/-*^ mice were characterized by altered microbiota composition, impaired intestinal clock, and microbiota rhythmicity irrespective of external clock disruption, which had no consistent colitis-promoting effect on *IL-10*^*-/-*^ mice. TRF delayed colitis onset reduced the expression of inflammatory markers and increased the expression of clock genes in the intestine, and increased gut microbiota rhythmicity in *IL-10*^*-/-*^ mice. Compositional changes and reduced rhythmicity of the fecal microbiota preceded colitis and could predict colitis symptoms for individual *IL-10*^*-/-*^ mice across different experiments. Our findings provide perspectives for new diagnostic and TRF-based, therapeutic applications in IBD that should be further explored.

## Introduction

Inflammatory bowel diseases (IBD), primarily Crohn’s disease and ulcerative colitis, comprise different chronic inflammatory conditions mostly of the colon but also other gastrointestinal sites, presenting with a range of mild to severe gastrointestinal problems and periods of flare-ups and remission. IBD prevalence is increasing worldwide, not only in so-called Western populations but also in newly industrialized countries of the Middle East, Asia, and South America.^1^ The specific etiology of IBD is unclear but thought to involve inflammatory interactions between the intestinal microbiome and the host’s immune system.^2^ Correspondingly, there is a need for better clinical options in IBD diagnosis, treatment and management, to predict IBD risk, onset, or flares or treat the underlying risk factors, root causes, and acute symptoms. IBD treatment is largely focussed on immunosuppression, including with steroids and biologics, such as anti-tumor necrosis factor (TNF) antibodies.^3^ Treatments targeted at the intestinal microbiota have also shown limited success in experimental trials, mostly in a fraction of treated patients, including with antibiotics and probiotics,^4^ fecal microbiota transplantation (FMT),^5^ or dietary interventions, such as exclusive enteral nutrition (EEN).^6^ However, as the underlying cause of the disease remains unknown, the optimal diagnostic and therapeutic targets for IBD prevention and treatment remain elusive.

The relative taxonomic composition of the intestinal (fecal) microbiota of mice and humans fluctuates with circadian rhythmicity,^7^ affecting metabolic and immune microbiome functions.^8,9^ Microbiota rhythmicity has been associated with the biological clock of the host, which is regulated by endogenous factors, including a central “master clock”, the suprachiasmatic nucleus (SCN) in the hypothalamus, and organ and cell-specific peripheral oscillators in different organs, including the intestines, as well as exogenous Zeitgebers, including light/dark cycles and dietary patterns.^10^ At the cellular level, the molecular clock consists of a transcription-translation feedback loop, whereby the ‘clock’ proteins circadian locomotor output cycles kaput (CLOCK) and brain and muscle ARNT-like (BMAL)-1 heterodimerize and mediate the transcription of a large number of genes, including *periods* (*Per1*, *Per2*, *Per3*) and *cryptochromes* (*Cry1*, *Cry2*), which are part of the negative feedback loop and inhibit CLOCK:BMAL-1-mediated transcription.^11^ Disruption of the biological clock by endogenous genetic or exogenous environmental factors can induce dysbiosis of the gut microbiome and intestinal inflammation in mice.^12,13^ In humans, shift work or jet lag have been associated with increased risks for obesity and diabetes,^10^ intestinal inflammation and IBD.^14^ Thus, dysbiosis of the gut microbiome as a consequence of clock disruption has been suggested as an etiological factor for the development of IBD,^12^ providing a new perspective for IBD treatment based on the restoration of biological clock and microbiota rhythmicity using interventions, such as intermittent fasting.^15^

Here, we study the relationship of IBD, biological clock, and gut microbiota based on the interleukin (*IL)-10*^*-/-*^ mouse model for experimental colitis, using altered (4 h/4 h) light–dark cycles and (8 h night) time-restricted feeding (TRF) to disrupt and restore circadian rhythmicity. We show circadian rhythmicity of the intestinal clock and gut microbiota to be impaired in *IL-10*^*-/-*^ mice irrespective of external clock disruption. We demonstrate inconsistent effects of clock disruption on experimental colitis, but extended survival, improved intestinal inflammation and gut microbiota rhythmicity in *IL-10*^*-/-*^ mice after TRF. Finally, we identify shifts in fecal microbiota composition and rhythmicity that precede colitis and correlate with the time of colitis symptom onset in individual mice. Our findings, if transferable to IBD patients, suggest new diagnostic and therapeutic applications that should be further studied.

## Methods

### Animal experiments

Unless stated otherwise, 8-week-old female BALB/cJ wild type (WT) mice [Jackson Labs, RRID:IMSR_JAX:000651] or IL-10 knockout (*IL-10*^*-/-*^) mice with a BALB/cJ background [RRID:IMSR_JAX:004333] were used for all experiments and kept in a specific pathogen-free barrier (SPF) facility under controlled conditions and a 12-h light and 12-h dark cycle. Both mouse lineages were bred for >15 generations as separate colonies in the central animal facility at the University of Hohenheim. The experiments were accredited by the Association for Assessment and Accreditation of Laboratory Animal Care. Mice were kept four to five mice in one cage, WT and IL-10^−/−^ mice were cohoused, they received a standard diet (ssniff Spezialdiäten GmbH, Soest, Germany) and drinking water *ad libitum*. All treatments and procedures were approved by the local Institutional Animal Care and Use Committee of the Ministry of Agriculture, Rural Areas, Veterinary and Food Sector of Stuttgart (permission number: RPS35-9185-99/396; RPS359-9185-99/406 permission received on 06/24/22; 01/16/23).

To analyze circadian clock gene expression in intestinal tissue, 10–15 weeks old male WT and *IL-10*^*-/-*^ mice, 32 animals each, were acclimatized for 2 weeks under a 12-h light and 12-h dark cycle and then half of the mice were exposed to a disrupted circadian rhythm for 2 weeks. Disruption of circadian rhythm was achieved using previously established models,^16,17^ i.e. by switching mice to an alternate light/dark cycle of 4-h light and 4-h darkness. Four mice per group were then sacrificed every 6 h at ZT3, ZT9, ZT15, and ZT21.

To investigate the effects of a disrupted circadian rhythm on onset of experimental colitis, mice were randomly divided into four groups, wildtype mice with an intact circadian rhythm of 12-h light and 12-h darkness (*n* = 8), wildtype mice with a disrupted circadian rhythm of 4-h light and 4-h darkness (*n* = 8), *IL-10*^*-/-*^ mice (*n* = 10) with an intact circadian rhythm and *IL-10*^*-/-*^ mice (*n* = 10) with a disrupted circadian rhythm. The condition was maintained for 12 weeks.

In addition, the influence of time-restricted feeding (TRF) on experimental colitis and disrupted circadian rhythm was investigated. Ten-week-old female mice were kept under a 12-h light and 12-h dark cycle for 2 weeks. Afterward the mice were divided into eight groups: *IL-10*^*-/-*^ mice with a 12 h/12 h cycle with food *ad libitum* (*n* = 10), *IL-10^−/−^* mice with a 12 h/12 h cycle and TRF (*n* = 10), *IL-10*^*-/-*^ mice with a 4 h/4 h cycle and food *ad libitum* (*n* = 10) and *IL-10*^*-/-*^ mice with a 4 h/4 h cycle and TRF (*n* = 10). Each group was also present with WT mice (*n* = 8). Disruption of circadian rhythm was achieved by switching the light/dark cycle to an interrupted alternate light/dark cycle of 4-h light and 4-h darkness. Mice that received TRF had access to food for 8 h from ZT16-ZT24. Experimental conditions lasted for 14 weeks. All mice had unlimited access to drinking water. The manifestation of colitis was monitored twice a day using the following clinical scores: (i) Change of body weight: 0, normal; 1, <10% weight loss; 2, 10–15% weight loss; 3, ≥15% weight loss. (ii) Consistency of stool: 0, normal; 1, softening of stool; 2, very soft stool; 3, liquid stool; (iii) Rectal inflammation: 0, no visible inflammation; 3, prolapse; (iv) Rectal bleeding: 0, no blood; 3, gross bleeding from the rectum. Mice had to be killed if one parameter alone reached a score of 3, or with an additive score ≥6, or with an additive score of 3–5, if the condition did not change within 24 h. In such cases of severe colitis or at the end of the experiment, mice were sacrificed through CO_2_ exposition and cervical dislocation. Colon tissue was removed and was in part stored with formalin for further histologic analyses and in part stored at −80 degrees for gene expression analyses.

### Histology

To analyze histological changes, 5 μm sections of colonic tissue samples fixed in 4% PBS buffered formalin solution and subsequently embedded in paraffin, were stained with hematoxylin and eosin (Carl Roth, Karlsruhe, Germany). The sections were analyzed and scored as previously described.^18^ Microscopic visualization of all parameters was conducted by usage of AxioVision software (Carl Zeiss Microscopy, Jena, Germany).

### RNA preparation and PCR analysis

Total RNA was isolated by using peqGold TriFast (VWR Life Science, Darmstadt, Germany). RNA concentration was determined by NanoDrop 2000c (Thermo Fisher Scientific GmbH, Erlangen, Germany), and 125 ng of total RNA was used for reverse transcription. Relative gene expression was analyzed by real-time polymerase chain reaction (qRT-PCR) and calculated using the ΔΔCT method. The housekeeping gene glyceraldehyde 3-phosphate dehydrogenase (GAPDH) was taken as a reference gene. Sequences of the specific sense/anti-sense primers for mouse:

*Gapdh*: 5′-TGT TCC TAC CCC CAA TGT GT-3′/5′-AGA GTG GGA GTT GCT GTT GA-3′; *Bmal-1*: 5′-GCC ACT GAC TAC CAA CTT GAT G-3′/5′-TGA TCC TTC CTT GGT GTT CTG C-3′; *Clock*: 5′-CCT AGA AAA TCT GGC AAA ATG TCA-3′/5′-CCT TTT CCA TAT TGC ATT AAG TGC *T*-3′; *Ccl-5*: 5′-GCT GCT TTG CCT ACC TCT CC-3′/5′-TCG AGT GAC AAA CAC GAC TGC-3′; *Cry1*: 5′-AGC CAG CTG ATG TAT TTC CCA-3′/5′-AGT TTA GTG ATG TTC CAT TCC TTG AA-3′; *Ifn-ɣ*: 5′-CTG ATG GGA GGA GAT GTC TA-3′/5′-CAC CAG GTG TCA AGT CTC TT-3′; *Per1*: 5′-CCG AAT ACA CAC TTC GAA ACC AG-3′/5′-TCC CGT TTG CAA CGC AG-3′; *Per2*: 5′-CTG GCT TCA CCA TGC CTG TT-3′/5′-AAG GCC TGA GGC AGG TTT G-3′; *Rev-erb α*: 5′-GTC TCT CCG TTG GCA TGT CT-3′/5′-CCA AGT TCA TGG CGC TCT 3′;* Tnf-ɑ*: 5′-GGA GGC AAC AAG GTA GAG-3′/5′-TGT CCA TTC CTG AGT TCT G-3′.

### DNA extraction from fecal samples

Bacterial DNA was extracted from fecal pellets using the Quick-DNA™ Fecal/Soil Microbe Miniprep Kit (Zymo Research, Hilden, USA). The samples were thawed on ice and weighed prior to being transferred to a BashingBead™ lysis tube containing 750 μl Bashing Bead Buffer. The samples were subjected to mechanical lysis through bead beating, which was performed five times for 45 s at a speed of 6 m/s using a FastPrep-24 5 G (MP Biomedicals, Eschwege, Germany). To prevent DNA damage from overheating, samples were incubated on ice for 5 min between each step. The subsequent steps were conducted in accordance with the instructions in the manual. The instructions were modified as follows: DNA fragments were washed twice with 500 μL gDNA Wash Buffer, and DNA elution with 50 μL DNA Elution Buffer was repeated twice. DNA concentration was determined using a NanoDrop™ One Microvolume UV-Vis Spectrophotometer (Thermo Fisher Scientific, Waltham, USA).

### Library preparation and sequencing

The Quick-16S™ NGS Library Prep Kit (Zymo Research, Hilden, USA) was used for library preparation of each of the four sequencing runs. The hypervariable V3-V4 region of the 16S rRNA gene was amplified using 2 μL of the DNA template. Besides DNA samples negative controls, comprising no template controls for the DNA extractions and blank controls for the PCR amplification, were employed. A ZymoBIOMICS Microbial Community DNA Standard (Zymo Research, Freiburg) was introduced as a positive control. Subsequent procedures were conducted in accordance with the instructions provided with the Quick-16S™ NGS Library Prep Kit (Zymo Research, Hilden, USA). The barcoded samples were normalized to a concentration of 30–45 ng, dependent on the run, and pooled. The DNA concentration of the library was determined using a Qubit 3.0 Fluorometer (Thermo Fisher Scientific, Waltham, USA). For sequencing, the library was denatured and diluted to a final concentration of 10 pM (run 1 and 2) or 11 pM (run 3 and 4). DNA sequencing was performed on an Illumina MiSeq using the 600-cycle MiSeq Reagent Kit v3 with 15% PhiX (Illumina, San Diego, CA, USA), in accordance with the manufacturer’s recommendations.

### Sequence processing

The raw sequencing data were processed using the open-source bioinformatics tool QIIME2 (Quantitative Insights into Microbial Ecology 2, version 2019.7). The DADA2 workflow was employed to remove primers and low-quality bases from the demultiplexed reads. Following the filtering, denoising, and merging of the reads, as well as the removal of chimeras, 77.45% (E.2) and 70.8% (E.3) of the raw reads were retained. Subsequently, amplicon sequence variants (ASVs) were generated. To account for the varying sequencing depths in diversity analyses, the sequences were rarefied to 9,007 (E.2) and 1,879 (E.3) reads per sample, respectively. For combined analysis of E.2 and E.3, all samples were rarefied to 2,425 sequences. For the differential abundance analysis, the rarefied ASV tables were taxonomically assigned at the genus level (L6) based on the Silva 132–99 database. The genus-level taxa counts of all samples are listed in Supplementary Table 1.

### Statistical analysis

Data visualization and statistical analysis were conducted using R (version 4.2.3.) and GraphPad Prism (version 10.3.1). The distribution of the data was evaluated using the Anderson-Darling and Shapiro–Wilk tests. Non-normally distributed parameters were examined using non-parametric tests, such as the pairwise Wilcoxon rank-sum test for group comparisons or Spearman’s rank correlation test for correlation analyses, while normally distributed parameters were analyzed using parametric tests, such as the pairwise t-test or two-way ANOVA with Tukey’s post-hoc multiple comparison test. For survival rates, Log-rank (Mantel-Cox) test and Gehan-Breslow-Wilcoxon test were used. The Benjamini–Hochberg procedure was employed to correct for false discovery rates. Unless otherwise stated, boxplots display medians and corresponding 95% confidence intervals (CI) along with significance thresholds of p/q > 0.05 ns, p/q < 0.05 *, p/q < 0.01 **, p/q < 0.001 ***, p/q < 0.0001 ****. n-values, representing the number of mice included in each test, are provided in the figure legends.

Beta-diversity analysis was conducted using the multidimensional Bray-Curtis Dissimilarity Matrix, which was reduced into a two-dimensional system and displayed in Principal Coordinate Analysis (PCoA) plots. The statistical testing of dissimilarities between sample groups was performed by calculating Analysis of Similarities (ANOSIM).

For the analysis of rhythmic oscillations of clock gene expression and relative abundance, a cosine wave regression analysis was applied.^19^ For clock gene expression, rhythmicity was measured per group whereas for microbiome data, measurements were taken separately for each mouse and taxon. Oscillations and their amplitudes were assessed over a 24-h period length and Benjamini-Hochberg -adjusted *p* values <0.05 were considered significant. The visualization of the diurnal profiles of significant rhythmic clock genes were visualized by fitting data points to the cosine-wave-curve-equation:

$$\mathrm{baseline}+\mathrm{amplitude}*\cos\left(2*\mathrm{pi}*\left(\mathrm{timepoint}-\mathrm{phaseshift}\right)\;/\;24\right).$$

Partial Least Squares Discriminant Analysis (PLS-DA) was conducted in order to predict the time until the first colitis symptoms and the day of sacrifice due to severe colitis symptoms based on microbiome profiles.^20^ Only taxa with a fecal relative abundance of >0.1% in at least three samples were considered; a pseudocount of 1 was added for zero values, and the resulting relative abundances were transformed using the centered log-ratio (clr) method. The models were trained on E.3 fecal relative abundance profiles from mice that were sacrificed during the experiment due to the development of severe colitis symptoms. The initial manifestation of loose stools was considered as the first colitis symptom, whereas the criteria for sacrifice resulted from a combination of very soft/diarrheal stool and weight loss as described before.^18^ Fecal samples from ZT3 at the baseline, week 2, and week 6 were included in the analysis. A Spearman’s rank correlation test was employed to assess the association between the predictions for mice from E.2 and E.3 and the observed time to the first symptoms or sacrifice. Moreover, based on the PLS-DA models, normalized coefficients were calculated for each taxon to identify taxa that contribute to a longer or shorter time until first colitis symptoms or sacrifice.

A metadata table that contains the background information about every sample that is needed to reproduce the result from the manuscript is provided in Supplementary Table 2. The QIIME commands and R code used for sequence processing and statistical analysis are listed in Supplementary Table 3. Additional statistical details, including p-values from all cosinor analyses, are described in Supplementary Table 4.

## Results

### *The intestinal circadian clock is disrupted in* IL-10^−/−^ *mice*

**Figure 1.**
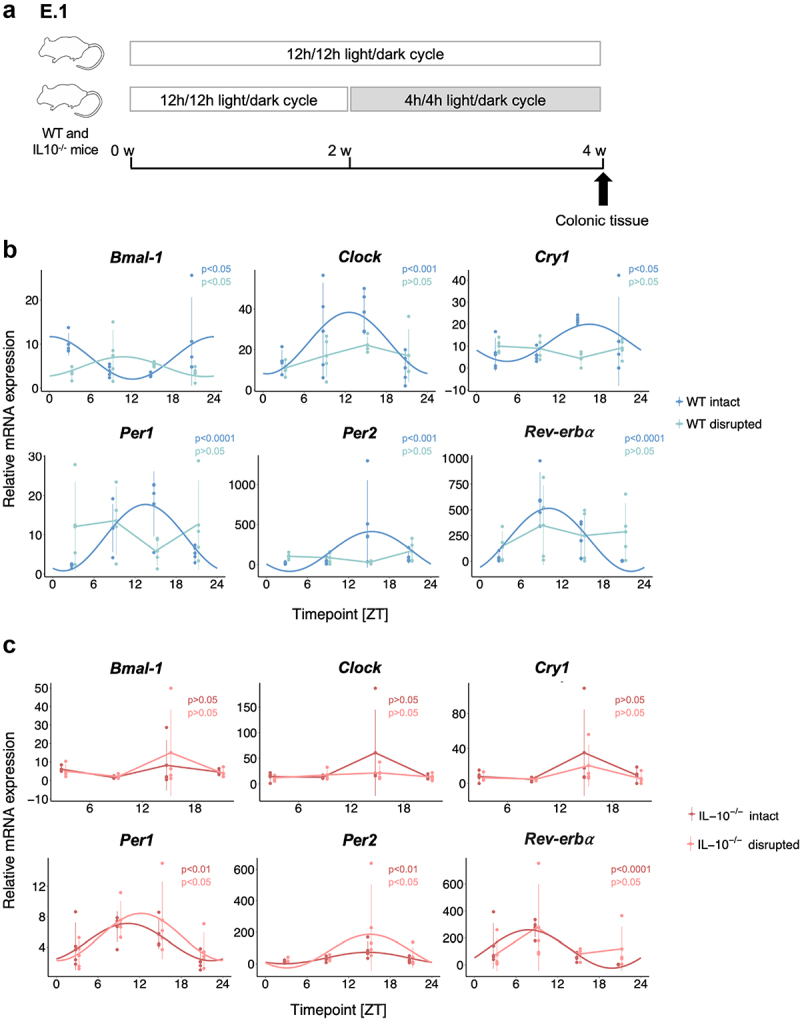
Intestinal circadian clock gene expression is disrupted in *IL-10*^*-/-*^ mice.

To study the relationship between the circadian clock, intestinal inflammation, and colitis, we used the *IL-10*^*-/-*^ mouse model for experimental colitis and altered light–dark cycles to disrupt circadian rhythmicity (E.1, Figure 1a). Individual *IL-10*^*-/-*^ and wildtype (WT) BALB/cJ mice from different litters were housed in separate cages and assigned to normal (12 h/12 h) or altered (4 h/4 h) light/dark cycles, with mice from all groups showing similar age profiles. All mice were sacrificed after 4 weeks and gene expression levels of *Bmal-1*, *Clock*, *Cry1*, *Per1*, *Per2*, and *Rev-erbα* determined in colon tissue RNA by quantitative real-time PCR. *IL-10*^*-/-*^ mice did not develop colitis symptoms during the experiment. Intestinal gene expression levels across all mice per group were tested for significant circadian rhythmicity with cosinor-based models (see Methods for details).

(A) 10–15-week-old male *IL-10*^*-/-*^ and WT mice (32 mice per group) were individually housed and exposed to normal (12 h/12 h) or altered (4 h/4 h) light/dark cycles. For clock gene expression analysis of WT (B) and *IL-10*^*-/-*^ (C) mice (*n* = 4 per group and time point), mice were sacrificed at 6 h intervals (ZT3, ZT9, ZT15, and ZT21) and colonic tissue was used for RNA extraction and gene expression analysis by qRT-PCR. The circadian rhythmicity of relative mRNA expression for *Bmal-1*, *Clock*, *Cry1*, *Per1*, *Per2*, and *Rev-erbα* in colonic tissue was measured by cosine-wave regression with cosinor-based techniques and Benjamini–Hochberg correction. Only the gene expression profiles with significant circadian rhythmicity (*p* < 0.05) are shown with smoothed curves.

In WT mice, intestinal gene expression of all clock genes showed significant rhythmicity under normal light/dark conditions, which was no longer detected under altered light/dark conditions (Figure 1b), confirming the general utility of our light-based clock disruption model, as previously demonstrated.^16,17^ In contrast, intestinal circadian rhythmicity was abolished even under normal light/dark conditions in *IL-10*^*-/-*^ mice for *Bmal-1*, *Clock*, *and Cry1* (Figure 1c), whereas *Per1, Per2*, and *Rev-erbα* exhibited rhythmicity, albeit at lower amplitude compared to WT mice (Suppl. Fig. S1). Circadian rhythmicity of intestinal *Rev-erbα* gene expression was diminished under altered light/dark conditions in *IL-10*^*-/-*^ mice similarly to WT mice (Figure 1c). Thus, the intestinal circadian clock is disrupted in IL-10 deficient mice, including asymptomatic mice that have not yet developed experimental colitis, suggesting a link between the susceptibility to colitis and the circadian clock in this model.

### *Light-induced clock disruption does not consistently affect colitis in* IL-10^*-/-*^ *mice*

**Figure 2.**
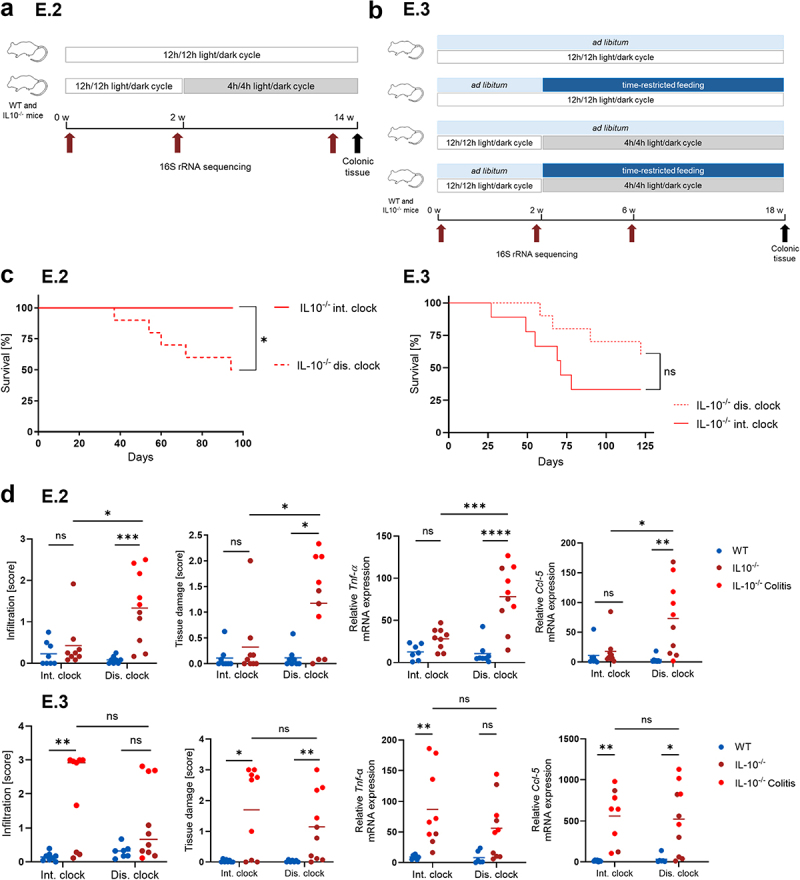
Light-induced clock disruption does not consistently affect colitis in *IL-10*^*-/-*^ mice.

To characterize the influence of clock disruption on the onset of experimental colitis in IL-10 deficient mice, 8-week-old *IL-10*^*-/-*^ and WT mice from different litters were co-housed in groups of 4–5 mice per cage for 2 weeks and subsequently exposed to 12 weeks of altered (4 h/4 h) light/dark cycles (E.2, Figure 2a). Fecal samples were collected before and at 2 and 14 weeks after cohousing and intestinal tissue samples were harvested at the end of the experiments or at the time of sacrifice for *IL-10*^*-/-*^ mice that developed severe colitis (see Methods for details). Taxonomic microbiota compositions were determined by 16S rRNA gene amplicon sequencing. In a second related experiment, in addition to external clock disruption, 8- to 12-week-old *IL-10*^*-/-*^ and WT mice were co-housed in groups of 4–5 mice per cage for 2 weeks and subjected to 16 weeks of time-restricted feeding (TRF) with food availability restricted to the last 8 h of the dark (active) phase, in order to test the effects of this intervention on colitis, clock and gut microbiota parameters (E.3, Figure 2b). Fecal samples were collected before and after the 2-week adaptation period and 4 weeks after clock disruption with or without TRF. Tissue samples were harvested as described for E.2.

Schematic illustrations of the experimental designs for (A) E.2: 8-week-old female *IL-10*^*-/-*^ (n = 20) and WT (n = 16) mice were co-housed and equally exposed to normal (12 h/12 h) or altered (4 h/4 h) light/dark cycles for 12 weeks and (B) E.3: 8–12-week-old female *IL-10*^*-/-*^ (n = 40) and WT (n = 32) mice were co-housed and equally exposed to normal or altered light/dark cycles, with either *ad libitum* feeding or time-restricted feeding for 16 weeks. (C) Survival rates of *IL-10*^*-/-*^ mice from E.2 (12 h/12 h n = 9, 4 h/4 h n = 10) and E.3 (12 h/12 h n = 9, 4 h/4 h n = 10). (D) Infiltration and tissue damage scores (E2: WT n = 8; *IL-10*^*-/-*^ n = 9/10; E3: WT n = 7/8; *IL10*^*-/-*^ n = 9/10) and relative *Ccl-5* (E2: WT n = 7/8; *IL10*^*-/-*^ n = 9/10; E3: WT n = 6/7; *IL10*^*-/-*^ n = 8/10) and *Tnf-ɑ* (E2: WT n = 7/8; *IL10*^*-/-*^ n = 9/10; E3: WT n = 6/7; *IL10*^*-/-*^ n = 9/10) mRNA expression in WT (blue) and *IL-10*^*-/-*^ mice. “IL-10^−/−^ Colitis” (light red) refers to mice killed before the end of the experiment due to severe colitis as defined with clinical scores (see Methods for details). Other *IL-10*^*-/-*^ mice without colitis symptoms (dark red) were sacrificed at the end of the experiment. For E.3, only *ad libitum*-fed mice are shown. Wilcoxon rank sum or two-way ANOVA with Tukey’s post-hoc multiple comparison test (*p* value: ns > 0.05; * <0.05; ** <0.01; *** <0.001; **** <0.0001). For survival curves, log-rank (Mantel-Cox) test and Gehan-Breslow-Wilcoxon test (*p* value: ns > 0.05; * <0.05) were used.

*Ad libitum* (AL)-fed *IL-10*^*-/-*^ mice exposed to altered light/dark cycles from E.2 but not E.3 developed experimental colitis earlier than *IL-10*^*-/-*^ mice under normal light/dark conditions (Figure 2c) and displayed increased colonic inflammation in histological scores (immune cell infiltration, tissue damage, bowel wall thickness) and gene expression levels of the proinflammatory cytokine TNF-*ɑ*, chemokine ligand (CCL)-5 and interferon (IFN)-*ɣ* (Figure 2d, Suppl. Fig. S2, S3). However, reflecting the dysfunctional immunoregulation of *IL-10*^*-/-*^ mice, inflammatory markers were generally increased in AL-fed *IL-10*^*-/-*^ compared to WT mice from both experiments and as expected, the highest levels of inflammation were detected in *IL-10*^*-/-*^ mice that had to be sacrificed with severe colitis before the end of the experiment (Figure 2d). In conclusion, while external clock disruption may promote experimental colitis in *IL-10*^*-/-*^ mice, other factors appear to induce colitis even in the absence of external clock disruption.

### IL-10 deficiency is associated with altered gut microbiota composition and disrupted gut microbiota rhythmicity

**Figure 3.**
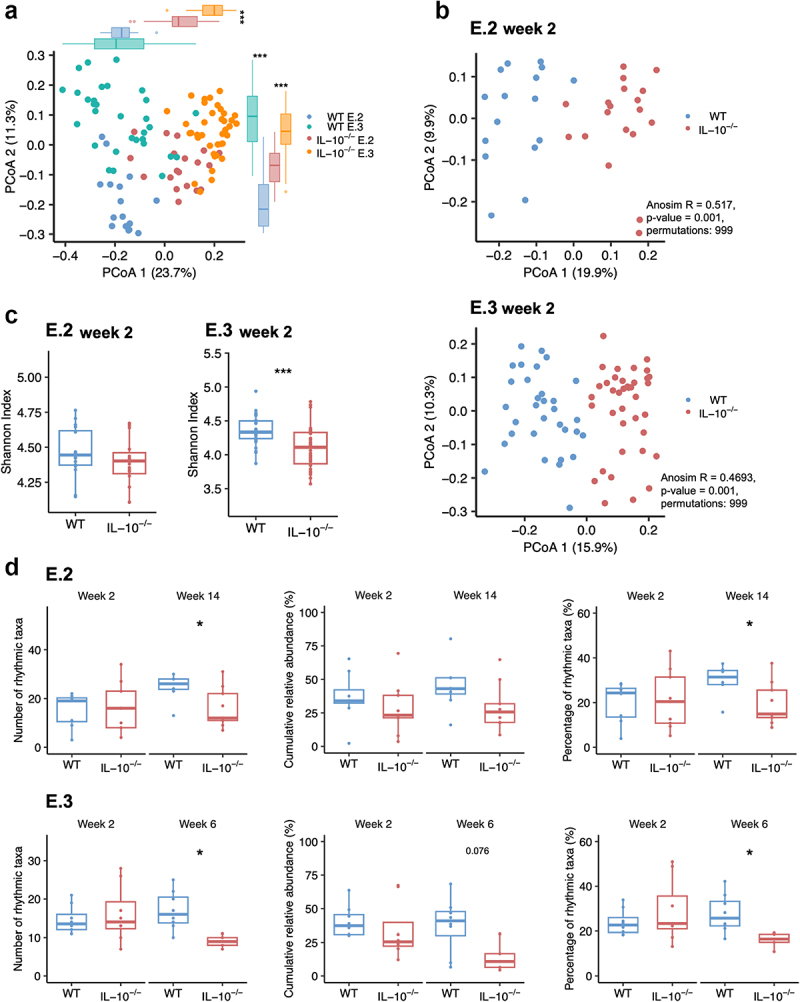
IL-10 deficiency is associated with altered gut microbiota composition and disrupted gut microbiota rhythmicity.

In both the E.2 and E.3 experiments, *IL-10*^*-/-*^ and WT mice showed marked compositional microbiota differences at ZT3, based on Bray-Curtis dissimilarity (Figure 3a), which persisted after cohousing (2 weeks, Figure 3b) and for the duration of the experiment (E.2: 14 weeks; E.3: 6 weeks, Suppl. Fig. S4) and were accompanied by reduced microbiota diversity (Shannon index) in *IL-10*^*-/-*^ compared to WT mice (Figure 3c). However, taxonomic microbiota compositions also differed between genetically identical mice from the two experiments (Figure 3a), highlighting potential experimental confounders.

Comparison of fecal microbial compositions based on Bray-Curtis dissimilarity between WT and *IL-10*^*-/-*^ mice from (A) all experiments or (B) split between E.2 (WT: n = 16; *IL-10*^*-/-*^: n = 19) and E.3 (WT: n = 31; *IL-10*^*-/-*^: n = 38). Mice were sampled 2 weeks after co-housing at the same time point (ZT3). Differences between groups along PCoA 1 and 2 were tested using Wilcoxon signed rank test with Benjamini–Hochberg correction (p-value: <0.001; ***). Significant clustering of samples was tested with ANOSIM. (C) Microbiota diversity per sample (Shannon index) for E.2 and E.3 of WT (blue) and *IL-10*^*-/-*^ (red) mice 2 weeks after co-housing (ZT3). (D) Microbiota rhythmicity was measured using relative abundance data per mouse and time point with cosine wave regression analysis with Benjamini–Hochberg correction. The number and percentage of rhythmic taxa and their cumulative relative abundance per mouse were compared separately for different time points and E.2 (Week 2, WT: n = 16; *IL-10*^*-/-*^: n = 19; week 14: WT: n = 8; *IL-10*^*-/-*^: n = 9) and E.3 (Week 2, WT: n = 32; *IL-10*^*-/-*^: n = 37; week 6, WT: n = 8; *IL-10*^*-/-*^: n = 6) including only AL-fed mice exposed to a normal (12 h/12 h) light/dark cycle. Wilcoxon rank sum or t-test with Benjamini–Hochberg correction (*p* value: ns > 0.05; * <0.05; ** <0.01; *** <0.001).

To measure gut microbiota rhythmicity, fecal samples were collected from individual mice at 6-h intervals, analogously to the intestinal clock gene expression analysis described above, at weeks 2 and 14 for E.2 and 2 and 6 for E.3 (Figure 2a,b). Cosinor-based models were used for cosine wave correlations of the relative abundances of bacterial taxa collected from individual mice over the course of sampling intervals (see Methods for details). On average, our analysis identified 18.86 ± 6.64 s.e.m rhythmic bacterial taxa per WT mouse (28.01 ± 9.37%), accounting for 41.42 ± 16.11% of the total microbiota (cumulative relative abundance) as undergoing significant circadian rhythmicity in AL-fed WT mice exposed to normal (12 h/12 h) light/dark cycles (Figure 3d). Under identical conditions circadian rhythmicity of the gut microbiota was disrupted in *IL-10*^*-/-*^ mice, but only at later time points, as evidenced by significantly reduced numbers and fractions of bacterial taxa with rhythmic behavior at weeks 14 (E.2) and 6 (E.3) compared to week 2, as well as a trend toward reduced cumulative relative abundance of these taxa at week 6 (*p* = 0.076, Figure 3d). This was accompanied by altered oscillations in the relative abundance of different genera throughout the day in *IL-10*^*-/-*^ mice compared to WT mice (Suppl. Fig. S5). Except for different microbiota compositions in E.2, clock disruption in *IL-10*^*-/-*^ mice exposed to altered light/dark cycles was not associated with significant differences in α or β-diversity or in the number or cumulative relative abundance of rhythmic taxa at earlier or later time points relative to *IL-10*^*-/-*^ mice exposed to normal conditions (Suppl. Fig. S6). Together, our findings indicate that while *IL-10*^*-/-*^ mice exhibit compositional microbiota differences relative to WT mice early in life, disruptions of microbiota rhythmicity developed later in life. However, both the disruption of the intestinal clock and the gut microbiota rhythmicity preceded the appearance of colitis symptoms.

### *Time-restricted feeding reduces experimental colitis and intestinal inflammation and restores microbiota rhythmicity in* IL-10^−/−^ *mice*

To assess the potential of TRF to restore disrupted intestinal clock and microbiota phenotypes and delay the development of intestinal inflammation and colitis, we compared *IL-10*^*-/-*^ and WT mice subjected to TRF or *ad libitum* (AL) feeding from E.3 (Figure 2b).

**Figure 4.**
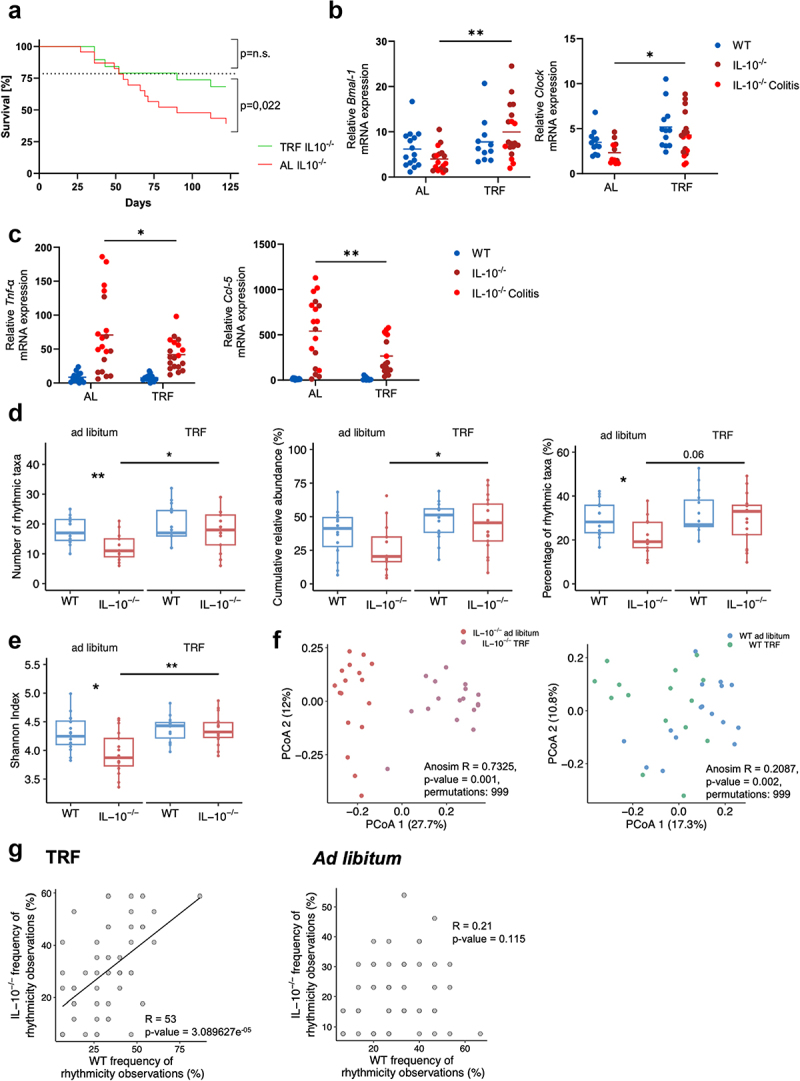
Time-restricted feeding reduces experimental colitis and inflammatory markers and restores the intestinal clock and microbiota rhythmicity in *IL-10*^*-/-*^ *mice*.

When the *IL-10*^*-/-*^ mice subjected to normal or altered light/dark cycles were studied separately, TRF-treated mice from both groups showed a non-significant trend toward longer survival compared to AL-fed mice (Suppl. Fig. 7). Since we had no detected discernible effects of the altered light/dark cycle on *IL-10*^*-/-*^ mice survival (Figure 2c), the intestinal clock (except for a disruption of *Rev-erbα*; Figure 1c), intestinal inflammation (Figure 2d), or gut microbiota rhythmicity (Suppl. Fig. S6), *IL-10*^*-/-*^ mice from both the normal and altered light/dark cycle groups were combined for the following analysis. Across *IL-10*^*-/-*^ mice from both groups, TRF was associated with increased survival after 52 days compared to AL (Figure 4a). As all mice were sacrificed at the same time (corresponding to ZT3 from Figure 1b,c), gene expression levels could be compared and showed increased expression of the clock genes *Bmal-1* and *Clock* (Figure 4b) and reduced expression of the inflammation markers *Tnf-ɑ* and *Ccl-5* (Figure 4c). TRF also restored the gut microbiota rhythmicity deficits of *IL-10*^*-/-*^ mice (Figure 3d), as TRF increased the number of rhythmic taxa and their cumulative relative abundance to the level of WT mice (Figure 4d). In addition, different relative abundance oscillation patterns were observed for individual bacterial taxa between TRF-treated and AL-fed *IL-10*^*-/-*^ mice (Suppl. Fig. 8). Similarly, TRF increased the microbiota α-diversity in TRF-treated compared to AL-fed *IL-10*^*-/-*^ mice to similar levels as seen in WT mice (Figure 4e). In both TRF-treated *IL-10*^*-/-*^ and WT mice, taxonomic microbiota compositions shifted, but more in *IL-10*^*-/-*^ mice (Figure 4f). To characterize the effects of TRF on specific microbiota members, we compared the frequency at which circadian rhythmicity was observed for individual bacterial genera across all mice. Rhythmicity was significantly more frequently observed for *Ruminoclostridium*, *Desulfovibrio*, *Blautia*, and *Alloprevotella* in TRF-treated compared to AL-fed *IL-10*^*-/-*^ mice, all of which also showed frequent rhythmic behavior in AL-fed WT but not *IL-10*^*-/-*^ mice (Suppl. Fig. S9). In fact, the frequency of rhythmic behavior of individual bacterial taxa positively correlated between TRF-treated but not AL-fed *IL-10*^*-/-*^ mice and AL-fed WT mice (Figure 4g), demonstrating that TRF increased the rhythmicity of the same bacteria in *IL-10*^*-/-*^ mice that show rhythmicity in WT mice.

(A) Survival rates of *IL-10*^*-/-*^ mice from E.3 subjected to AL-feeding (n = 19) or TRF (n = 19), compared using log-rank (Mantel-Cox) and Gehan-Breslow-Wilcoxon test (*p* value: ns > 0.05). (B) Increased relative mRNA expression of *Bmal-1* (WT AL: n = 15, TRF n = 11; *IL-10*^*-/-*^ AL: n = 17, TRF: n = 19) and *Clock* (WT AL/TRF: n = 12; *IL-10*^*-/-*^ AL: n = 13, TRF: n = 18) and (C) decreased relative mRNA expression of *Tnf-ɑ* (WT AL: n = 13, TRF n = 13; *IL-10*^*-/-*^ AL: n = 19, TRF: n = 19) and *Ccl-5* (WT AL: n = 12, TRF n = 11; *IL-10*^*-/-*^ AL: n = 18, TRF: n = 17) in WT mice (blue) compared to *IL-10*^*-/-*^ mice with (light red) or without (dark red) colitis, defined with clinical scores as severe colitis and therefore sacrificed before the end of the experiment. (D) The number and percentage of rhythmic taxa and their cumulative relative abundance per mouse were compared between AL-fed and TRF-treated WT (blue; AL: n = 15; TRF: n = 15) and *IL-10*^*-/-*^ (red; AL: n = 13; TRF: n = 17) mice based on cosine wave regression with Benjamini–Hochberg correction. Differences in (E) microbiota diversity (Shannon, t-test) and (F) microbiota composition (Bray-Curtis dissimilarity, ANOSIM) at ZT3 AL-fed and TRF-treated WT (AL: n = 16; TRF: n = 15) and *IL-10*^*-/-*^ (AL: n = 17; TRF: n = 17) mice. (G) The frequency at which individual bacterial taxa were observed to show rhythmicity is positively correlated between AL-fed WT mice TRF-treated but not AL-fed *IL-10*^*-/-*^ mice. Spearman’s rank correlation: *p* value: ns > 0.05; **** <0.0001. Mice with disrupted and intact light/dark cycles were combined for all analyses. P-values based on Wilcoxon rank sum or t-test with Benjamini–Hochberg correction or two-way ANOVA with Tukey’s post-hoc multiple comparison test (*p* value: ns > 0.05; * <0.05; ** <0.01).

In summary, positive effects of TRF on survival and intestinal inflammation were associated with improved intestinal clock gene expression, and restored gut microbiota rhythmicity and diversity to the level of WT mice.

### *Colitis onset may be predictable for individual* IL-10^−/−^ *mice based on gut microbiota composition and rhythmicity*

**Figure 5.**
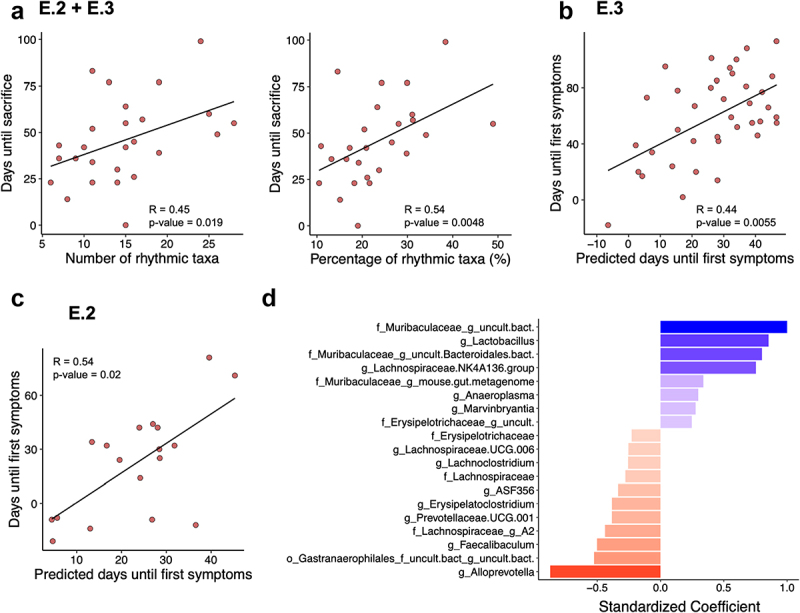
Colitis onset may be predictable for individual *IL-10*^*-/-*^ mice based on gut microbiota composition and rhythmicity.

As the disrupted rhythmicity and altered composition of the gut microbiota preceded the development of colitis and were detected in asymptomatic *IL-10*^*-/-*^ mice, we speculated that the developmental trajectory of individual mice toward the onset of colitis could be reflected in continuous, linear changes of these microbiota phenotypes. To test for a continuous relationship between gut microbiota changes and colitis development, all AL-fed *IL-10*^*-/-*^ mice that had to be sacrificed at different time points before the end of the experiments from E.2 and E.3 were combined, including mice subjected to normal and altered light/dark conditions. Both the number of bacterial taxa with significant circadian rhythmicity per fecal sample (*R* = 0.45, *p* = 0.019) and the fraction that they represented of all detected taxa (*R* = 0.54, *p* = 0.005) positively correlated to the remaining lifetime of the corresponding *IL-10*^*-/-*^ mouse, before it had to be removed from the experiments (Figure 5a), suggesting that the disruption of gut microbiota rhythmicity is a linear process that is linked to colitis development.

(A) The remaining time in days until individual AL-fed *IL-10*^*-/-*^ mice had to be sacrificed due to colitis was positively correlated to the number and percentage of rhythmic taxa in their fecal microbiota (E.2 + E.3; n = 35). (B) Predictions for the remaining time until *IL-10*^*-/-*^ mice developed loose stools as the first colitis symptom, based on a partial least squares discriminant analysis model trained on fecal microbiota profiles from E.3 mice (n = 38), correlated positively with the actual observed days. (C) Predictions from the same model performed comparably well when tested on *IL-10*^*-/-*^ mice from E.2 (n = 18). Spearman’s rank correlation: *p* value: * < 0.05; ** <0.01. (D) Normalized coefficients of the most informative taxa from the E.3-trained model that are associated with a longer (blue) or shorter (red) time until mice developed colitis symptoms.

Next, we tested for correlations between taxonomic fecal microbiota compositions and the first appearance of loose stool colitis symptoms. This parameter allowed us to increase the number of datapoints for the statistical analysis, as several *IL-10*^*-/-*^ mice developed mild or transient colitis symptoms without having to be sacrificed. For this, partial least squares discriminant analysis (PLS-DA) was used on the fecal relative abundance profiles of all bacterial taxa as observable variables and the remaining time of the corresponding *IL-10*^*-/-*^ mouse to the first appearance of colitis symptoms as the predicted variable (Figure 5b). When trained on all available samples from E.3, the predictive model showed a good fit to the data (*R* = 0.44, *p* = 0.0055) and, more importantly, the model predictions matched the observed time to first symptoms (*R* = 0.54, *p* = 0.02) when tested on the independent data from the available samples of E.2 (Figure 5c). Training and testing of a model with the remaining time to sacrifice for the subset of *IL-10*^*-/-*^ mice that developed colitis during the E.2/3 experiments as an alternative predictive variable generated comparable results (E.3 training data: *R* = 0.45, *p* = 0.002, E.2 test data: *R* = 0.53, *p* = 0.024; Suppl. Fig. S10), suggesting robust associations between microbiota composition and colitis development despite underlying compositional microbiota differences between *IL-10*^*-/-*^ mice from different experiments (Figure 3a). Two *Muribaculaceae* genera, *Lactobacillus*, and a *Lachnospiraceae* genus were identified as the most informative taxa correlated with longer and *Alloprevotella* as the most informative taxon correlated with shorter symptom-free survival by the model (Figure 5d). In summary, our findings suggest that the assessment of gut microbiota rhythmicity and composition has the potential to predict the colitis development of *IL-10*^*-/-*^ mice. However, altered gut microbiota rhythmicity and composition may reflect independent colitis associations, as bacterial taxa that were most informative for the predictive colitis model (Figure 5d) generally did not overlap with those that showed increased rhythmicity after TRF (Suppl. Fig. 9), which improved colitis in our experiments.

## Discussion

IBD, long thought of as a problem of industrialized countries, has also been increasing in newly “westernized” regions of the world,^21^ suggesting that disease development, in addition to genetic risk factors, is also influenced by environmental and lifestyle parameters.^22,23^ Compositional and functional microbiota alterations that characterize IBD have sparked considerable scientific and clinical interest.^24,25^ However, to what extent the microbiome may determine IBD development or merely reflect intestinal ecosystem changes that stem from the disease remains controversial,^26^ resulting in uncertainty about the degree to which specific lifestyle interventions, such as altered dietary habits, could promote, prevent, or treat IBD.

The gut microbiota composition of mice and humans undergoes alterations with circadian rhythmicity that, when disturbed by internal or external Zeitgebers, can have profound metabolic consequences, including insulin resistance, obesity, and metabolic syndrome.^27,28^ There is also increasing evidence that links immune functions and IBD to the intestinal circadian clock, since immune cells exhibit diurnal oscillations in gene expression and effector functions, which are disturbed by a disruption of the circadian clock and impaired in IBD patients.^29,30^ In mice, the disruption of the light/dark cycles promotes gut leakiness^31^ and the epithelial cell proliferation is reduced in *BMAL-1* deficient mice,^30^ whereas the expression of clock genes in mucosal tissue and blood cells is decreased in IBD patients, even in non-inflamed regions of the large intestine.^32^ Our findings of abolished rhythmicity and reduced amplitude of intestinal circadian clock gene expression (*Bmal-1*, *Clock*) in *IL-10*^*-/-*^ mice demonstrate that experimental colitis is accompanied by a dysfunctional intestinal clock, in line with another recent study.^12^ In humans, clock disruption has been described as an early manifestation of IBD.^32^ Our experiments provide less conclusive evidence for a direct colitis-promoting role of external clock disruption (4 h/4 h light/dark cycles). While external clock disruption impaired the intestinal clock gene expression of WT mice, symptom-free *IL-10*^*-/-*^ mice exhibited comparable impairments even under normal light/dark conditions (Figure 1). There was, however, no evidence for additional clock-disruptive or pro-inflammatory effects in *IL-10*^*-/-*^ mice after external clock perturbation, which also did not consistently increase development of colitis or reduce survival in *IL-10*^*-/-*^ mice. This contrasts with recent findings by Niu et al., who reported colitis-promoting effects in mice with a genetic, epithelium-specific clock deficiency (*Bmal-1*^*IEC−/−*^), using either DSS-induced mice or genetically susceptible (*Bmal-1*^*IEC−/−*^x*IL-10*^*−/−*^) double knockout mice.^12^ These different observations could indicate stronger and broader disruptive effects of the *Bmal1*-dependent genetic clock deficiency on the intestinal inflammatory and metabolic milieu compared to those induced by altered light/dark cycles. However, it should be noted that colonization of germ-free *Bmal-1*^*IEC−/−*^ mice with cecal contents of inflamed *IL-10*^*-/-*^ mice but not clock- deficient *Bmal-1*^*IEC−/−*^ mice induced inflammatory immune responses,^12^ suggesting that primarily IL-10 deficiency induced dysbiosis of the gut microbiome with colitis-promoting effects on the murine host. Importantly, the fecal microbiota of *IL-10*^*-/-*^ mice from our experiments consistently displayed distinct taxonomic compositions and an increasingly disrupted circadian compositional microbiota rhythmicity, even before the development of colitis symptoms (Figure 3). Thus, while the specific relationship between intestinal clock and colitis development in *IL-10*^*-/-*^ mice remains unclear, a better understanding of the compositional and rhythmic microbiome changes that precede colitis symptoms may help to identify predictive markers for disease development.

Lifestyle influences have not only been discussed as detrimental factors for IBD development but, in the context of intermittent fasting or time-restricted eating, also as external dietary Zeitgebers to sustain the circadian clock.^33^ Predominantly applied to control weight loss and improve metabolic disorders, such as metabolic syndrome and type 2 diabetes,^34^ intermittent fasting (IF) has also been associated with immunomodulatory functions, including the downregulation of pro-inflammatory mechanisms involved in IBD,^35^ such as decreased plasma concentration of TNF-α and IL-8 in non-obese patients after 5 weeks of TRF.^36^ Using a TRF model with feed access limited to the last 8 h of the active (dark) phase, we observed significant positive effects on *IL-10*^*-/-*^ mice, irrespective of whether these mice were subjected to normal or altered light/dark conditions. TRF-treated *IL-10*^*-/-*^ mice survived longer, showed lower levels of inflammatory markers and improved rhythmicity of intestinal circadian clock gene expression. TRF also increased the fecal microbiota rhythmicity of *IL-10*^*-/-*^ mice compared to *ad libitum*-fed mice, restoring levels of clock gene expression and microbiota rhythmicity to those of WT mice. Using a slightly different 12 h night time TRF approach, Niu et al. showed similar positive effects, which were dependent on a functional clock, as no improvements were observed after TRF in *Bmal-1*^*IEC−/−*^x*IL-10*^*−/−*^ double knockout mice.^12^ Combined, these two studies provide strong evidence that TRF can compensate to some extent for the disruptive effects of IL-10 deficiency on intestinal clock and experimental colitis. Clinical data on the benefits of intermittent fasting or time-restricted eating for IBD patients is lacking.^15^ Ramadan fasting, which in contrast to the mouse experiments discussed here, limits human food consumption to the inactive dark phase, has in one study been associated with worsening clinical parameters.^37^ Yet, the increasing evidence for metabolic benefits of intermittent fasting and the great affordability and accessibility of this dietary intervention to patients warrant further studies on its therapeutic potential for IBD prevention and management.

Experimental colitis in *IL-10*^*−/−*^ mice is multifactorial but microbiota-dependent,^38^ as germ-free *IL-10*^*−/−*^ mice do not develop colitis^39^ and colitis can be attenuated or aggravated by colonizing germ-free or specific pathogen-free mice with different bacterial isolates, such as *Lactobacillus* and *Helicobacter* species.^40^ Murine gut microbiota compositions can vary considerably between animals, including mice from the IL-10 deficient lineage that has been continuously bred for multiple generations in our facility (Figure 3b). The timeline and presentation of colitis symptoms can therefore be difficult to control and predict for individual *IL-10*^*-/-*^ mice, not unlike the onset and development of IBD in individual patients.^41^ We show that reproducible changes in microbiota rhythmicity and composition precede the appearance of colitis symptoms in our IL-10 deficient mice. The number of bacterial taxa undergoing circadian rhythmic changes in relative abundance was negatively correlated to the remaining survival time of individual *IL-10*^*−/−*^ mice. Based on individual fecal taxonomic microbiota profiles, both the remaining survival time and the time until the first appearance of loose stools could be reproducibly predicted, using observations from one experiment as a training set and from another experiment as a test set. Our findings therefore indicate that the disruption of intestinal microbiota rhythmicity and transition to microbiome dysbiosis is a continuous process that begins before colitis onset and that microbiota changes underlying this colitis developmental process are conserved, at least in our experimental setting. The ability to predict experimental colitis development for individual *IL-10*^*−/−*^ mice could make this IBD mouse model easier to handle and manipulate for functional and mechanistic microbiome studies. Moreover, if translatable to the human IBD setting, comparable prediction models could improve IBD risk stratification and help IBD patients with an earlier introduction of therapy and a better disease management.^42^

Our study has several limitations: (i) Our analyses could have been confounded by underlying microbiota differences between WT and *IL-10*^*−/−*^ mice, although both lineages have continuously been bred for >15 generations at our animal facilities, and mice from both lineages and different litters were randomly cohoused for 2 weeks before each experiment. (ii) Measurements of intestinal clock gene expression in male mice and microbiota rhythmicity and responses to time-restricted feeding in female mice could have been influenced by sex differences. However, disrupted intestinal clock gene expression patterns similar to our observations in male mice have previously been reported for female mice^17^ and colonic *Bmal-1* expression at ZT3 showed no significant difference between male mice from E.1 and female mice from E.3 (data not shown). Moreover, other findings related to microbiota rhythmicity, response to TRF and microbiota-based colitis prediction were shown in female mice independently of potential sex differences. (iii) Our findings indicate impaired intestinal clock gene expression in non-colitic *IL-10*^*−/−*^ mice even without light-induced clock disruption. While phenotypes of impaired microbiota rhythmicity in the same mice would also suggest that colitis onset may be preceded by a disruption of intestinal clock functions, our analyses are insufficiently powered to exclude low-amplitude intestinal clock gene expression rhythmicity and, thus, basic intestinal clock functionalities in these mice. These questions as well as others about the inconsistent effects of clock disruption on colitis in the *IL-10*^*−/−*^ mice should be further studied in the future.

Using the IL-10 deficient experimental colitis mouse model, our study sheds new light on the relationship between IBD, the circadian clock and the gut microbiome, demonstrating that a disrupted intestinal clock and circadian rhythmicity of the gut microbiota reflect dysbiosis in the course of colitis development, which can be mitigated with TRF. These findings suggest clinical relevance of gut microbiota rhythmicity and composition as potential biomarkers with predictive potential for colitis development and of TRF as a promising dietary intervention to prevent colitis in high-risk individuals, which should be further studied.

## Supplementary Material

Supplemental Material

## Data Availability

16S rRNA gene amplicon sequence data is available at the European Nucleotide Archive (ENA; https://www.ebi.ac.uk/ena/) under the accession number PRJEB82312. All additional information that is needed to reproduce the presented findings is included with the publication and its supplemental material.
